# Antitumor effects and mechanisms of traditional Chinese medicine gamboge: A review

**DOI:** 10.3389/fphar.2025.1650560

**Published:** 2025-08-18

**Authors:** Yanqing Zhou, Jialing Chen, Qin Zhu, Binyan Lin

**Affiliations:** ^1^ School of Pharmacy, Nanjing University of Chinese Medicine, Nanjing, China; ^2^ School of Basic Medicine and Clinical Pharmacy, China Pharmaceutical University, Nanjing, China; ^3^ Experiment Center of Science and Technology, Nanjing University of Chinese Medicine, Nanjing, China

**Keywords:** gamboge, antitumor, gambogic acid, gambogenic acid, traditional Chinese medicine

## Abstract

Traditional Chinese medicine (TCM) gamboge is a dried resin obtained from *Garcinia hanburyi* Hook f. For over 500 years, TCM gamboge has been used to treat scrofula, carbuncle, jaundice, furuncle, and other chronic and stubborn diseases. An increasing amount of evidence has proven the significant anticancer properties of the main active ingredients from gamboge in recent years. The ingredients of gamboge, such as gambogic acid (GA) and gambogenic acid (GNA), can inhibit tumor growth through various processes, including apoptosis induction, cell cycle arrest, tumor cell invasion and migration inhibition, and autophagy regulation. In this review, we elaborate on the role of the main active ingredients of gamboge in treating cancers. It would be enlightening to provide the possible therapeutic applications of gamboge in the clinic.

## 1 Introduction

Cancer is one of the leading causes of death worldwide, with a high incidence and mortality rate. As estimated by the American Cancer Society, there are 1,958,310 new cancer cases, and 609,820 cancer deaths are anticipated to occur in the United States in 2023 (Siegel et al., 2023). Surgery, radiotherapy, conventional chemotherapy, hormone therapy, immunotherapy, and targeted therapies are the main clinical treatment ways for cancer treatment. Additional therapy methods are also explored and applied. However, drug-related toxicities such as hair loss; heart, kidney, or nerve toxicity; infertility; and drug resistance caused additional challenges (Haque et al., 2021). Therefore, it is urgent to find an effective but low-toxicity drug. Many traditional Chinese medicines (TCMs) have been recorded in Chinese antiquarian books for their effectiveness in treating canker sores and erysipelas. These diseases represent inflammation or cancer in contemporary medical science. Moreover, a variety of TCMs (Wang Y. et al., 2020), including *Rhodiola rosea* (Loo et al., 2010; Rong et al., 2020), *Astragalus membranaceus* (Auyeung et al., 2016), *Coptis chinensis* Franch (Iizuka et al., 2000; Liu L. et al., 2020), *Garcinia hanburyi* Hook f. (Hahnvajanawong et al., 2010; Anantachoke et al., 2012; Yang et al., 2013), and *Tripterygium wilfordii* (Yu et al., 2020), have been proven to treat different types of cancers. Natural compounds are characterized by their multiple targets and low toxicity (Luo et al., 2019). Meanwhile, new targets can be found based on natural products. Therefore, natural compounds from TCMs should receive increased attention for cancer treatment.

The main sources of natural compounds are terrestrial plants, marine macro-organisms, and micro-organisms from the sea and land, characterized by their wide range of sources, structural diversity, and low toxicity. Natural compounds have been demonstrated to have a broad potential curative value for the therapy of various cancers, including lung cancer (Oh et al., 2019; Zhao et al., 2022), liver cancer (Anwanwan et al., 2020; Zheng et al., 2021), stomach cancer (Chen et al., 2020b), breast cancer (Küpeli Akkol et al., 2022; Malla et al., 2022), and colorectal cancer (Rejhová et al., 2018; Sanchez-Martin et al., 2022). Anticancer drugs such as paclitaxel, vincristine, and doxorubicin (DOX) are derived from natural organisms. Natural compounds usually affect multiple molecular targets, such as transcription factors, cytokines, chemokines, adhesion molecules, growth factor receptors, and inflammatory enzymes (Haque et al., 2021). Moreover, natural compounds have been proven to improve patient survival rates by increasing cancer cell sensitivity and reducing or reversing resistance to chemotherapy drugs (Rejhová et al., 2018; Maleki Dana et al., 2022; Sanchez-Martin et al., 2022). Therefore, the role of natural compounds in cancer therapy cannot be ignored.

**FIGURE 1:**
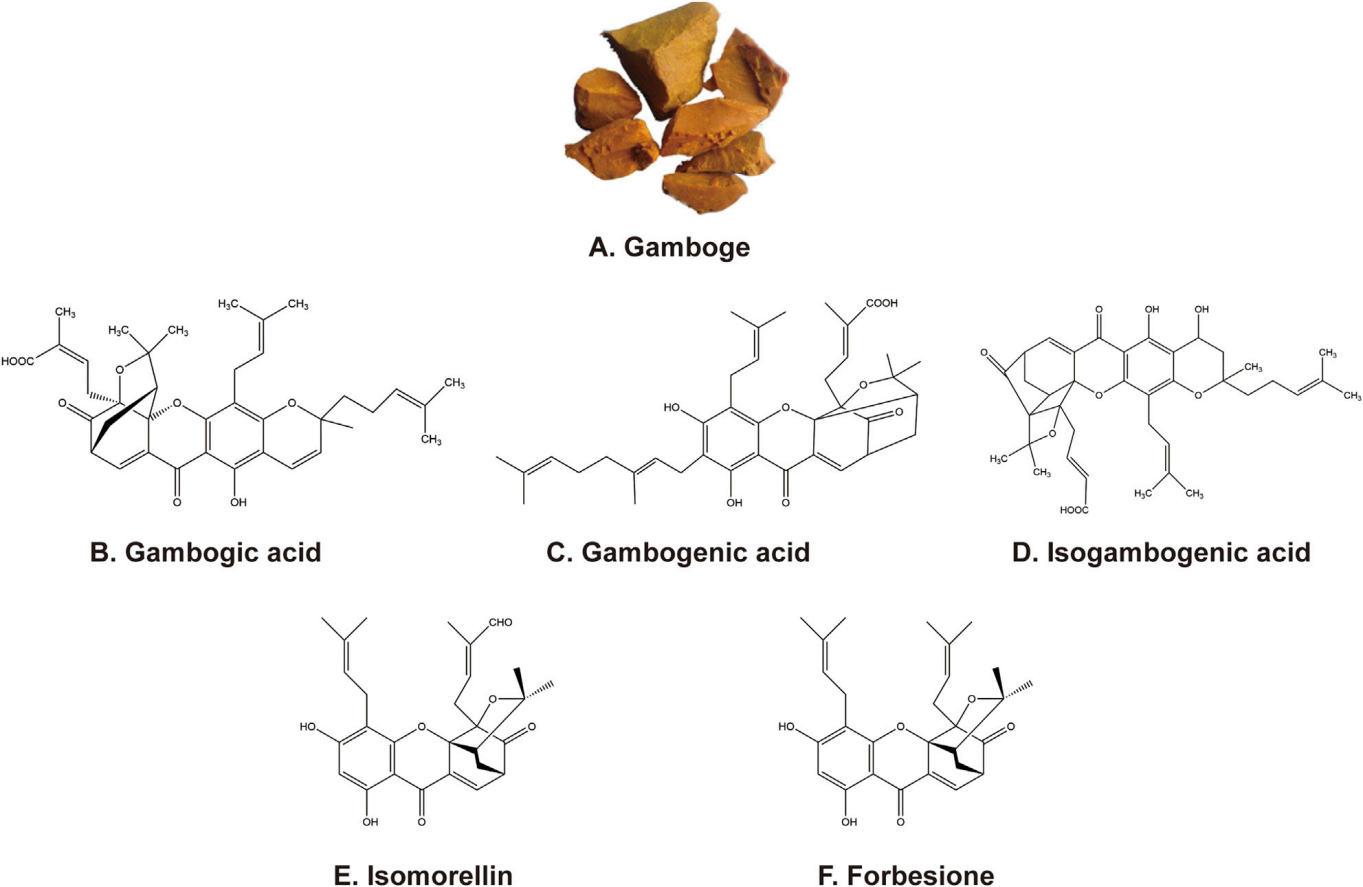
**(A)** Appearance of gamboge. **(B–F)** Chemical structure of the five active ingredients from gamboge.

Chinese medicine gamboge (Figure 1A) is a reddish yellow/orange-yellow colloidal resin secreted by *Garcinia hanburyi* Hook f., mainly from China, Cambodia, Thailand, Vietnam, India, and other tropical regions. Since ancient times, gamboge has been used to treat scrofula, carbuncle, and boils, which modern medicine considers to be inflammation or cancer. Several caged xanthones are isolated from gamboge have been reported to have antitumor activities (Gold-Smith et al., 2016; Zhao et al., 2022). The main active ingredients from gamboge include gambogic acid (GA) (Figure 1B), gambogenic acid (GNA) (Figure 1C), isogambogenic acid (iso-GNA) (Figure 1D), isomorellin (Figure 1E), and forbesione (Figure 1F). Multiple reports validated that these components inhibit tumor cells in different pathways (Hahnvajanawong et al., 2010; Anantachoke et al., 2012; Yang et al., 2013). In this review, we aim to summarize the antitumor research process of the main active xanthone ingredients from Chinese medicine gamboge and to improve the progress of these compounds in preclinical studies.

## 2 Gambogic acid

**FIGURE 2:**
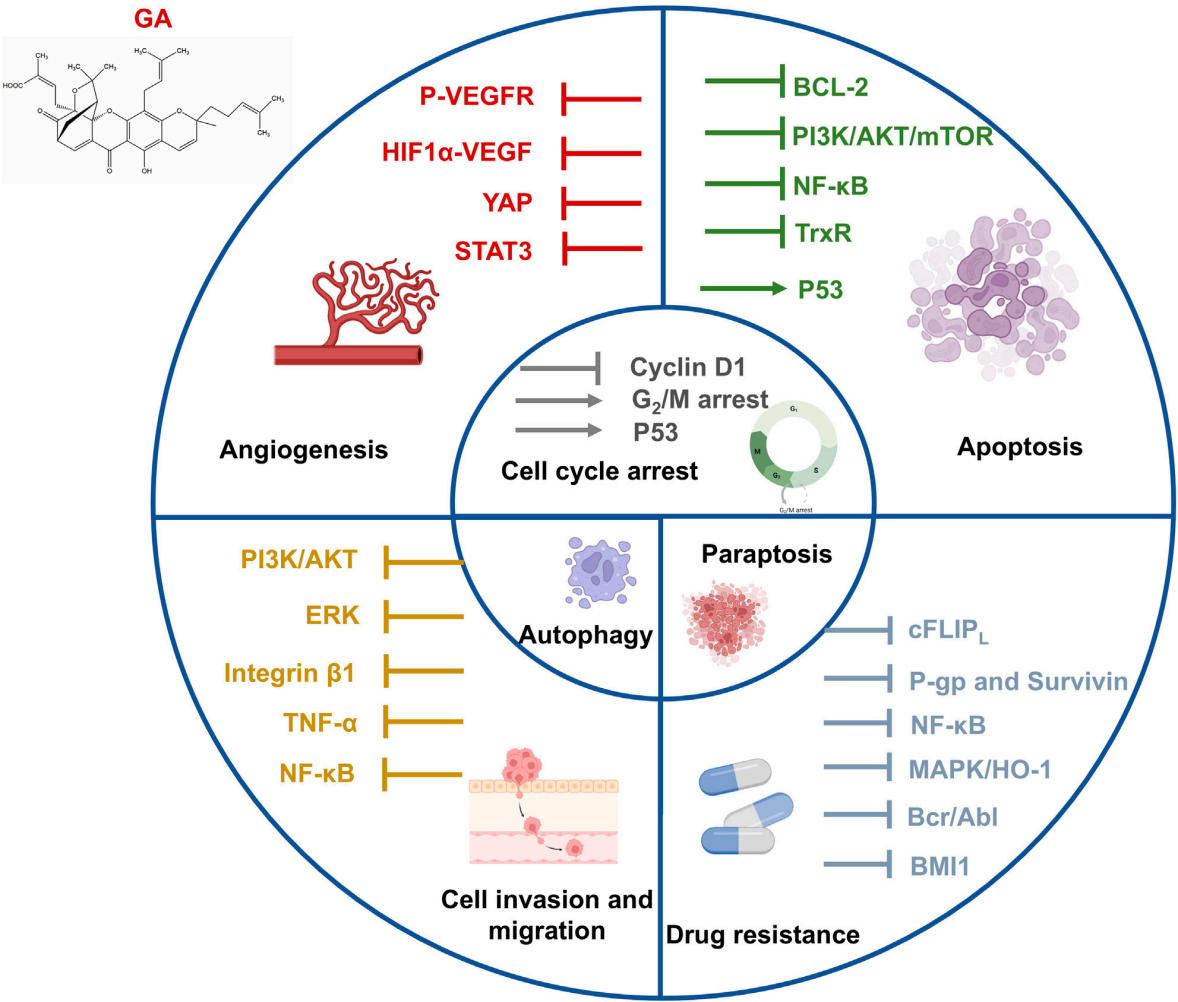
Antitumor mechanisms of gamboge active ingredient gambogic acid.

GA, one of the principal active ingredients in gamboge, is a caged xanthone compound with various bioactivities. GA prevents the development of tumors by inducing apoptosis, regulating cell autophagy, blocking the cell cycle, restricting cell metastasis, and impeding angiogenesis. Antitumor effects of GA in different types of cancer, including lung, breast, liver, pancreatic, and colorectal cancers, have been illustrated through *in vitro*/*in vivo* experiments (Hatami et al., 2020a; Liu Y. et al., 2020). Here, we summarize the antitumor effects of GA from multiple mechanisms and applications (Figure 2).

### 2.1 Antitumor mechanisms of GA

#### 2.1.1 Apoptosis

Apoptosis is recognized as a programmed cell death that occurs through both intrinsic (mitochondrial pathway) and extrinsic (death receptor pathway) processes. Apoptosis can be triggered by cellular stress, genetic damage, and the binding of ligands to death receptors (Pistritto et al., 2016). Mitochondrial outer membrane permeabilization (MOMP) is a key step during cell apoptosis. The proapoptotic members of the B-cell lymphoma (BCL)-2 family proteins such as BAK induce or promote MOMP, whereas the antiapoptotic BCL-2 proteins interrupt MOMP occurrence (Gupta et al., 2009). The intrinsic apoptotic pathway is activated by intracellular signals (including imbalanced homeostasis, intense oxidative stress, and DNA damage) to activate the cell death program (Lemke et al., 2014). GA is known as an antagonist of antiapoptotic BCL-2 family proteins (Zhai et al., 2008). GA antagonized BCL-2 family proteins, activated BAX/BAK, and promoted the release of apoptotic proteins such as cytochrome c and AIF into the cytoplasm, leading to the formation of apoptosomes and activation of caspases (O'Neill et al., 2016; Singh et al., 2019; Popgeorgiev et al., 2020). The transferrin receptor (TfR) is a target for cancer immunotherapy and also a target protein of GA. The attachment of GA and TfR triggered the apoptosis of tumor cells probably through the mitochondrial pathway (Kasibhatla et al., 2005; Ortiz-Sánchez et al., 2009). Excess reactive oxygen species (ROS) production induces an intrinsic apoptosis pathway in tumor cells (Zou et al., 2017). GA combined with thioredoxin reductase (TrxR) induced the imbalance of antioxidant defense, leading to the accumulation of intracellular ROS, which resulted in intracellular thiol depletion and oxidative stress that killed tumor cells (Duan et al., 2014).

The extrinsic apoptotic pathway depends on activating tumor necrosis factor (TNF) family death receptors by immune cells or receptor-activating drugs (Pollak et al., 2021). The PI3K/Akt/mTOR pathway is one of the most commonly triggered pathways in cancer cells, which plays various roles in normal physiological and carcinogenic processes, including cell proliferation, survival, and differentiation (Beck et al., 2014). The engagement of E-cadherin enhances the activation of DR4 and DR5 proapoptotic receptors, thereby promoting the progression of apoptosis (Singh and Lim, 2022). GA upregulated the expression of E-cadherin while blocking the mTOR signaling pathway to inhibit cell proliferation (Li X. et al., 2019).

P53, a key tumor suppressor, is one of the most frequently mutated proteins in cancer which suppresses the growth of tumors by triggering cell cycle arrest, cellular senescence, apoptosis, and genetic damage repair (Zhao and Sanyal, 2022). The murine double minute 2 (MDM2) gene encodes a p53 negative regulator. GA enhanced the expression of p53 by downregulating the transcription of MDM2, which inhibited the combination of MDM2 and p53, leading to the apoptosis of tumor cells (Gu et al., 2008).

Aberrant activation of NF-κB is related to various cellular processes in cancer, including cell proliferation, metastasis, angiogenesis, chemotherapy, and radiotherapy (Aggarwal and Sung, 2011). GA blocked the NF-κB signaling by targeting G protein-coupled receptor 108 (GPR108) in pancreatic and colorectal cancers (Lyu et al., 2022). GA triggered apoptosis in Burkitt’s lymphoma Raji cells by upregulating death-inducer obliterator 1 (DIO-1) and downregulating NF-κB and Bcl-xL (Yang and Chen, 2013). In addition, GA downregulated the expression of cellular FADD-like inhibitory protein (cFLIP) L and induced apoptosis in renal carcinoma Caki cells, probably through the inhibition of the NF-κB pathway (Jang et al., 2016). Table 1 shows IC_50_ of GA in a variety of cancer cell lines *in vitro*.

**TABLE 1:** IC_50_ of active ingredients in gamboge for different types of cancer cell lines.

Compound	Type of cancer	Cell	IC_50_	Treatment time (h)	Reference
Gambogic acid	Melanoma	A375	2.86 μM 2.15 μM 1.55 μM	24 48 72	Li et al. (2019a)
		B16-F10	2.61 μM 1.89 μM 1.26 μM	24 48 72	
	Chronic myeloid leukemia	KBM5	0.32 μM	48	Shi et al. (2014)
		KBM5-T315I	0.35 μM	48	
		K562	0.40 μM	48	
	Breast cancer	MDA-MB 453	1.5 μM	24	Seo et al. (2019)
		MDA-MB 468	2.35 μM	24	
		MDA-MB 435S	1.33 μM	24	
		MCF7-ERα-Y537S	1.81 ± 0.21 μM	96	Liu et al. (2021)
		MCF-7	1 μM	48	Wang et al. (2015)
		MCF-7	4.11 μM	48	Gu et al. (2008)
	Non-small-cell lung cancer	A549	3.56 ± 0.36 μM	48	Wang et al. (2014b)
		NCI-H460	4.05 ± 0.51 μM	48	
		NCI-H1299	1.12 ± 0.31 μM	48	
	Colorectal cancer	HCT-15P	1.08 μM	48	Wen et al. (2015)
		HCT-15R	0.87 μM	48	
		HCT116	1.1 µM 0.6 µM 0.5 µM	12 24 36	Zhang et al. (2014)
		HCT116	1.24 μM	48	Gu et al. (2008)
	Pancreatic cancer	PANC-1	7.198 μM 3.780 μM 0.977 μM	12 24 48	Xia et al. (2017)
		BxPC-3	2.692 μM 0.362 μM 0.778 μM	12 24 48	
		MIA PaCa-2	4.520 μM 2.721 μM 1.635 μM	12 24 48	
		SW1990	8.204 μM 3.055 μM 0.795 μM	12 24 48	
	Cervical carcinoma	HeLa	4.17 ± 0.30 μM 2.19 ± 0.11 μM 1.59 ± 0.05 μM	24 48 72	Feng et al. (2016)
		HeLa	3.53 μM	48	Gu et al. (2008)
	Cholangiocarcinoma	KKU-100	3.22 ± 0.17 μM 2.83 ± 0.18 μM 2.47 ± 0.03 μM	——	Hahnvajanawong et al. (2012)
		KKU-M156	1.96 ± 0.01 μM 1.30 ± 0.15 μM 0.76 ± 0.14 µM	——	
	Hepatoma	HepG2	3.8 μM	48	Gu et al. (2008)
	Cervical carcinoma	HeLa	4.17 ± 0.30 μM 2.19 ± 0.11 μM 1.59 ± 0.05 μM	24 48 72	Feng et al. (2016)
		HeLa	3.53 μM	48	Gu et al. (2008)
	Cholangiocarcinoma	KKU-100	3.22 ± 0.17 μM 2.83 ± 0.18 μM 2.47 ± 0.03 μM	——	Hahnvajanawong et al. (2012)
		KKU-M156	1.96 ± 0.01 μM 1.30 ± 0.15 μM 0.76 ± 0.14 µM	——	
	Hepatoma	HepG2	3.8 μM	48	Gu et al. (2008)
Gambogenic acid	Hepatoma	HepG2	3.23 μM 2.62 μM 2.14 μM	24 48 72	Yan et al. (2012)
		HepG2	2.141 μM	48	Xu et al. (2019a)
		HepG2/ADR	4.532 μM	48	
		HepG2	4.4 ± 0.12 μM	48	Yuan et al. (2016)
		HepG2	10.71 μM	24	Tang et al. (2018)
		SMMC-7721	4.25 ± 0.14 μM	48	Luo et al. (2015)
		NCI-H446	1.4 µM	——	Huang et al. (2019)
		NCI-H1688	2.4 µM	——	
	Colorectal cancer	HCT116	1.88 μM 1.48 μM	24 48	Zhao et al. (2020)
		SW620	2.83 μM 1.81 μM	24 48	
		DLD-1	1.97 μM 1.55 μM	24 48	
		SW480	8.159 μM	24	Li et al. (2022a)
		HCT116	8.172 μM	24	
	Nasopharyngeal carcinoma	CNE-2Z	2.25 μM 1.33 μM	24 48	Su et al. (2019)
		CNE-1	1.87 μM	72	Yan et al. (2011)
	Melanoma	A375	2.875 μM	24	Wang et al. (2022b)
		A2058	1.263 μM	24	
		B16	2.085 μM	24	
		B16F10	0.9959 μM	24	
	Non-small-cell lung cancer	HCC827	1.51 μM	72	Xu et al. (2018)
		HCC827ER	1.328 μM	72	
		H1650	0.909 μM	72	
		A549	7 μM	48	Yu et al. (2012)
		HCC827	1–2 μM	——	
		NCI-H1975	2–3 μM	——	
		A549	8.07 μM	24	Tang et al. (2018)
	Breast cancer	4T1	7.57 μM	24	Wang et al. (2021)
	Gastric cancer	SGC-7901	16.15 μM	24	Tang et al. (2018)
Isogambogenic acid	Glioma	U251 U-87MG	3–4 µM	24	Zhao et al. (2017)
	Cervical carcinoma	HeLa	6.35 µM	——	Yang et al. (2013)
	Non-small-cell lung cancer	A549	12.69 µM	——	
	Colorectal cancer	HCT-116	11.74 µM	——	
	Hepatoma	HepG-2	6.35 µM	——	
Isomorellin	Cholangiocarcinoma	KKU-100	3.46 ± 0.19 μM 3.78 ± 0.02 μM 4.01 ± 0.01 µM	24 48 72	Hahnvajanawong et al. (2021)
		KKU-100	6.2 ± 0.13 μM 5.1 ± 0.11 μM 3.5 ± 0.25 µM	24 48 72	Hahnvajanawong et al. (2012)
		KKU-M156	1.9 ± 0.22 μM 1.7 ± 0.14 μM 1.5 ± 0.14 µM	24 48 72	
		KKU-100	3.34 ± 0.12 μM	72	Hahnvajanawong et al. (2014)
		KKU-M139	2.71 ± 0.10 μM	72	
		KKU-M156	2.26 ± 0.05 μM	72	
Forbesione	Cholangiocarcinoma	Ham-1	3.34 ± 0.31 μM	72	Boueroy et al. (2017)
		KKU-100	3.53 ± 0.05 μM	72	Hahnvajanawong et al. (2014)
		KKU-M139	2.29 ± 0.04 μM	72	
		KKU-M156	2.63 ± 0.05 μM	72	
Ethanolic extract of gamboge	Colon cancer	SW480-GFP	0.54 μg/mL 0.36 μg/mL 0.24 μg/mL	24 48 72	Wang et al. (2018b)
		Ham-1	3.34 ± 0.31 μM	72	Boueroy et al. (2017)
		KKU-100	3.53 ± 0.05 μM	72	Hahnvajanawong et al. (2014)

#### 2.1.2 Cell cycle arrest

Cell cycle arrest is a well-known anticancer mechanism. GA reduced the level of cyclin D1 protein while increasing p53 expression to induce G_1_ phase arrest in human colorectal cancer cells *in vitro* (Wen et al., 2015). GA treatment induced G_2_/M phase arrest in human nasopharyngeal carcinoma (NPC) CNE-2 and 5-8F cells (Ren et al., 2022). In addition, Feng et al. found that GA can suppress the growth of human cervical carcinoma HeLa cells by increasing the amount of the G_2_/M phase (Feng et al., 2016).

#### 2.1.3 Cell invasion and migration

Tumor cell invasion and migration are labels of cancer development that allow tumor cells to escape from normal developmental regulation (Geho et al., 2005). Cell adhesion to the extracellular matrix (ECM) is critical in the cancer metastasis cascade. GA inhibited the migration and adhesion of malignant melanoma cells via suppressing the PI3K/Akt and ERK signaling pathways *in vitro* (Li C. Y. et al., 2019). GA suppressed integrin β1 and the membrane lipid raft-associated integrin signaling pathway to inhibit breast tumor cell adhesion *in vitro* (Li et al., 2011). Moreover, GA may restrain TNF-α-induced migration and invasion by blocking PI3K/Akt and NF-κB signaling pathways in human prostate cancer PC3 cells (Lü et al., 2012).

#### 2.1.4 Angiogenesis

Angiogenesis is a well-known hallmark of cancer that provides necessary oxygen and nutrients for tumor growth. The most common angiogenesis inducer is vascular endothelial growth factor (VEGF)-A (Hanahan and Weinberg, 2000). The activation of vascular endothelial growth factor receptor 2 (VEGFR2) by the VEGF is the primary factor driving tumor angiogenesis (Vimalraj, 2022). GA inhibited the tube formation of human umbilical vein endothelial cells (HUVECs) and reduced the level of phospho-VEGFR2 in melanoma cells *in vitro* (Li C. Y. et al., 2019). GA can reduce HIF-1α/VEGF expression *in vivo* to suppress tumor angiogenesis, suggesting that GA might be a new potential drug to treat human multiple myeloma (Wang F. et al., 2014). In addition, GA restricted VEGF-induced angiogenesis by diminishing the YAP nuclear expression in a dose-dependent manner *in vitro*/*in vivo*, leading to the inactivation of downstream STAT3 in HUVECs (Wan et al., 2019).

#### 2.1.5 Autophagy

Autophagy is a double-edged sword in regulating the tumor growth. It is widely accepted that autophagy suppresses tumor initiation, but evidence suggested that autophagy processes in established tumors are required to support uncontrolled cell growth for tumor maintenance. In breast cancer, loss of *BECN1*, an autophagy-associated gene, results in tumor-prone conditions (Liang et al., 1999). On the other hand, some tumor tissues exhibit high levels of LC3 puncta and lipidated LC3, supporting the role of autophagy in maintaining pancreatic cancer development (Fujii et al., 2008). P53 mutation was detected in the vast majority of malignancies, which may lead to an increase in the oncogenic activity (Duffy et al., 2017). Mutant p53 inhibits autophagy by blocking AMPK and activating the AKT/mTOR pathway through overexpression of growth factor receptors (Khromova et al., 2009; Cordani et al., 2016). Autophagy induction can deplete mutant p53 protein to interfere with cancer development (Choudhury et al., 2013). GA promoted mutp53 degradation and tumor cell death by inducing autophagy (Foggetti et al., 2017). Similarly, inhibiting GA-induced autophagy in pancreatic cancer cells can enhance its proapoptotic function (Wang et al., 2019). Suppression of GA-induced cytoprotective autophagy promotes apoptosis in colorectal cancer (CRC) cells. Evidence showed that GA-induced autophagy might be involved in tumor growth suppression *in vivo*. The result revealed a new perspective on GA in CRC treatment, which may be necessary in combination with autophagy inhibitors (Zhang et al., 2014).

#### 2.1.6 Drug resistance

Resistance to chemotherapeutic agents is a primary obstacle in cancer treatment. Numerous studies have confirmed that GA has the capacity to reduce drug resistance in tumor cells, making them more susceptible to chemotherapeutic drugs. GA can sensitize TNF-related apoptosis-inducing ligand (TRAIL)-mediated renal carcinoma Caki cell apoptosis via downregulating cFLIP_L_ (Jang et al., 2016), and it also sensitizes TRAIL-resistant breast cancer cells to TRAIL-induced apoptosis (Wang S. et al., 2018). P-glycoprotein (P-gp) and survivin expression were related to cancer multidrug resistance (Deng et al., 2021). GA elevated the susceptibility of DOX in drug-resistant breast cancer MCF-7/ADR cells by downregulating P-gp and survivin (Wang et al., 2015). GA also inhibited the NF-κB and MAPK/HO-1 pathways to enhance apoptosis triggered by cisplatin (CDDP) in non-small-cell lung cancer (NSCLC) (Wang L. H. et al., 2014). The activation of Bcr-Abl tyrosine kinase has been regarded as a characteristic of chronic myeloid leukemia (CML). GA deregulated the expression of Bcr-Abl and induced apoptosis in primary imatinib-resistant monocytes from patients (Shi et al., 2014). Glioma stem cells (GSCs) are strongly associated with high drug resistance in glioblastoma (GBM) (Boyd et al., 2021). Biotin-GA directly targeted the ring finger structural domain of B-cell-specific Moloney leukemia virus insert site 1 (BMI1), inducing BMI1 degradation and inhibiting the self-renewal capability of GSCs. Meanwhile, combining GA with temozolomide (TMZ) showed superior anti-GBM ability (Sun et al., 2024).

#### 2.1.7 Paraptosis

Paraptosis is a non-apoptotic cell death characterized by the lack of caspase inhibitor effects, cytoplasmic vacuolization, and mitochondrial swelling (Sperandio et al., 2000). Seo et al. (2019) observed that GA-induced cell death was accompanied by vacuolation and showed morphological and biochemical characteristics of paraptosis in breast cancer cells.

As mentioned above, multiple pieces of evidence have confirmed the antitumor effect of GA in a variety of cancers by inducing apoptosis and non-apoptosis cell death, arresting the cell cycle, inhibiting migration and invasion, angiogenesis, regulating autophagy, and reducing cellular drug resistance. Thus, GA might be the ideal agent for cancer therapy.

### 2.2 Nanoscale drug delivery system

GA has shown potent antitumor activity with clinical significance. However, its clinical application is limited due to its poor aqueous solubility, instability, low bioavailability, and severe systemic toxicity. Different types of nanoscale drug delivery systems, such as micelles, nanoparticles, and liposomes, have been applied to solve these challenges (Liu Y. et al., 2020).

In recent years, polymeric micelles have been used widely in preclinical studies. GA encapsulated by a multiple environment-sensitive prodrug self-assembled micelles based on chitosan graftomer increased the release and distribution in tumor tissue significantly compared to free GA, resulting in improved GA tumor-targeting ability (Du et al., 2021). Cai et al. (2014) prepared micelles formed by condensation of low-molecular-weight monomethoxy-poly (ethylene glycol) (mPEG)-2000 with GA, which showed 2.7 × 10^5^ times higher aqueous solubility than that of GA and decreased the poisonous side effect of GA effectively. Redox/pH dual-responsive and magnetic targeted hybrid multifunctional complex micelles (SPEG/HA/CSO-SS-HEX/Fe_3_O_4_/GA) were developed as a drug delivery system for GA to improve triple-negative breast cancer (TNBC) therapeutic efficacy. The tumor suppression rate *in vivo* of SPEG/HA/CSO-SS-HEX/Fe_3_O_4_/GA was 84.1%, which was 2.19 times higher than that of GA (Sang et al., 2018). Treating with free GA and GA-loaded PEG-pHis-PLGA/TPGS micelle system resulted in a significant decrease in P-gp in MCF-7/ADR cells, but the mixed micelle was better. The result suggested that the micelle system may be a viable strategy for GA to overcome clinical drug resistance in breast cancer (Wang et al., 2015).

Wang et al. prepared GA-loaded nanobubble–microbubble complexes (GA/PLGA-CMB) that could be used to open the blood–brain barrier noninvasively and reversibly under the action of focused ultrasound (FUS). GA/PLGA-CMB also supported GA to be distributed uniformly throughout tumor tissue for targeted glioma therapy (Wang F. et al., 2022). Both GA-loaded biomimetic nanoparticles (RBCm-GA/PLGA NPs) and GA could induce S phase arrest in CRC SW480 cells *in vitro*, but the RBCm-GA/PLGA group markedly reduced the tumor volume and relative tumor volume *in vivo* compared with the GA group (Zhang et al., 2017).

The liposome delivery system is widely used in tumor treatment, and liposomes have been the most successful drug delivery carriers among the nanoparticles studied (Natarajan et al., 2014). The repression of Bcl-2 was 1.23-fold higher after treatment with positively charged PEGylated liposomal formulation of GA (GAL) *in vitro* than that with free GA (Doddapaneni et al., 2016). Dang et al. (2021) prepared CB5005N-GA-liposome using the thin film hydration method, which showed a nearly three times higher percentage of tumor growth inhibition in breast cancer cells *in vivo* than that of GA-Sol.

The introduction of GA (GPgWSC) copolymer by polyethylenimine (PEI)-grafted water-soluble chitosan (WSC) achieved target specificity via targeting tumor cells overexpressing TfR (Park et al., 2022).

### 2.3 Applications in cancers

#### 2.3.1 Breast cancer

Breast cancer is one of the most common cancers and the most frequent malignancies in women. Current therapeutic options include surgery, chemotherapy, radiotherapy, adjuvant treatment, and target therapy (Harbeck and Gnant, 2017; Li et al., 2022b). However, poor prognosis, drug resistance, and high relapse risk of breast cancer indicate that it is essential to seek a novel drug to treat breast cancer. GA has shown its ability to increase the apoptosis rate significantly via combination with other antitumor agents. Optimized protein-fragment complementation assay revealed that GA acts as an antagonist of estrogen receptor alpha (ERα) Y537S. GA directly targeted ERα Y537S and inhibited MDA-MB-231 cells with the ERα Y537S mutant, inducing MCF7 cell apoptosis combined with CDK4/6 inhibitor abemaciclib (Liu et al., 2021). MCF-7/ADR cells showed an increase in the sub-G_1_ phase (23.15%) and apoptosis rate (19.7%) after 48 h GA (1 μM) treatment. The accumulation of the sub-G_1_ phase increased to 41.95%, and the number of apoptotic nuclei and annexin V-PI-positive cells increased to 38.6% after being treated with DOX and GA concurrently, possibly resulting from sensitizing MCF-7/ADR cells to DOX by inhibiting P-gp and survivin (Wang et al., 2015). In addition, treatment with TRAIL (25 ng/mL) and GA (0.25 μM) induced 14.7% and 13.8% apoptosis, respectively, in MCF-7 cells. However, concurrent TRAIL and GA treatment increased the apoptosis rate to 51.8%, which was associated with enhanced sensitivity of MCF-7 cells to TRAIL by GA (Wang S. et al., 2018).

#### 2.3.2 Non-small-cell lung cancer

Lung cancer is still a leading cause of cancer death, accounting for 23% of all cancer deaths. NSCLC accounts for 40%–45% of all cases of lung cancer (Desai et al., 2023). Liver kinase B1 (LKB1) is a tumor inhibitor that mediates cellular functions and is one of the most frequently mutated genes in NSCLC (Shukuya et al., 2019). GA exhibited stronger inhibitory effects in cells with wild-type LKB1 than that with mutated LKB1 cells. GA upregulated the level of p-AMPK by enhancing the binding of E-cadherin to LKB1 while suppressing the Akt/mTOR signaling pathway (Li X. et al., 2019). Gemcitabine (Gem) is considered a first-line option for NSCLC patients (Wu et al., 2014). However, the therapeutic effect of Gem is hampered by drug resistance (Olaussen and Postel-Vinay, 2016). GA reduced Gem resistance and promoted Gem antitumor potential *in vitro*/*in vivo*. The IC_50_ of Gem was reduced to 4.4, 2.2, and 0.63 nM by treatment with 100, 200, and 400 nM GA concurrently in A549 cells. A similar trend was observed in H1299 cells. The GA + Gem group showed minimal proliferating cell nuclear antigen (PCNA) marker staining (27%) compared to control (95%), GA (86%), and Gem (42%) treatments *in vivo*, indicating that tumor cell proliferation was suppressed by the GA + Gem group. These results confirmed the synergistic action of the GA and Gem combination in NSCLC (Hatami et al., 2020b). GA increased the accumulation of ROS via inhibiting CDDP-induced upregulation of HO-1. The tumor inhibition rate *in vivo* was 69.3% by treatment with GA and CDDP concurrently, whereas those treated with GA and CDDP alone were 29.0% and 57.2%, respectively, suggesting that the combination of GA and CDDP may provide a potential regimen to treat NSCLC (Wang L. H. et al., 2014).

#### 2.3.3 Colorectal cancer

CRC is the fourth most deadly cancer all over the world. It is the women’s second most common cancer and the men’s third (Dekker et al., 2019). After treatment with GA for 48 h, the level of proapoptotic proteins increased significantly in HT-29 cells. Moreover, the tumor volume in the tumor xenograft mouse model was decreased in a dose- and time-dependent manner after treatment with GA (Huang et al., 2015). Wen et al. (2015) indicated that GA activated the c-Jun N-terminal kinase (JNK) signaling pathway and induced apoptosis in both 5-fluorouracil (5-FU)-sensitive and 5-FU-resistant cells, suggesting that GA had the potential to combat 5-FU resistance in CRC. The IC_50_ value of GA in HCT116 cells was 1.1, 0.6, and 0.5 µM for 12, 24, and 36 h, respectively. In addition, GA induced protective autophagy, thereby restricting its antitumor effects via increasing 5-LOX-regulated ROS levels (Zhang et al., 2014).

#### 2.3.4 Pancreatic cancer

Pancreatic cancer is a deadly cancer that is predicted to be the second leading cause of cancer-related death before 2040 (Halbrook et al., 2023). GA promoted the accumulation of autophagosomes, inducing protective autophagy in both PANC-1 and BxPC-3 cells, which resulted from activating the autophagic promoter (Beclin-1) by inhibiting the Akt/mTOR pathway. However, concurrent treatment with GA and chloroquine (CQ) led to excessive accumulation of ROS, then triggered oxidative stress, resulting in apoptosis, and exhibited the strongest antitumor efficacy *in vivo* (Wang et al., 2019). The inhibition of the ERK/E2F1 signaling pathway by GA induced apoptosis in pancreatic cancer cell lines, reducing ribonucleotide reductase subunit-M2 (RRM2) expression. The tumor inhibition rate was 72.9% in the combined group (GA and Gem) compared to the control group, indicating that GA can promote the sensitivity of pancreatic cancer cells to Gem (Xia et al., 2017).

#### 2.3.5 Gastric cancer

Gastric cancer (GC) has poor survival with limited treatment; therefore, GC remains a leading cause of cancer-related mortality worldwide. GA showed a concentration-dependent inhibition of GC cell growth accompanied by apoptosis, oxidative DNA damage, and autophagy induction (Joha et al., 2023). MicroRNA (miRNA)-driven post-transcriptional gene silencing regulates biological processes, including cell proliferation, apoptosis, and development. GA induced ferroptosis in GC through the miR-1291/FOXA2 axis (Qian et al., 2025) and GA induced apoptosis via the circRNA_ASAP2/miR33a-5p/CDK7 axis (Lin et al., 2020). In addition, GA contributed to docetaxel resistance reversion in GC by inhibiting survivin (Wang et al., 2008). In conclusion, GA showed anti-GC effects through multiple processes.

#### 2.3.6 Other cancers

GA exhibited IC_50_ in human cervical carcinoma HeLa cells as 4.17 ± 0.30, 2.19 ± 0.11, and 1.59 ± 0.05 µM for 24, 48, and 72 h treatment, respectively, and it exhibited a dose-dependent increase in the number of cells in the G2/M phase (Feng et al., 2016). GA induced G2/M phase arrest and apoptosis in CNE-2 and 5-8F cells. Moreover, GA inhibited the overexpression of CD47 stimulated by chemotherapy drugs and showed a synergistic effect with 5-FU (Ren et al., 2022).

### 2.4 Toxicity and clinical application

GA has demonstrated good antitumor efficacy in preclinical studies. Nevertheless, its side effects and poor hydrophilicity have limited its clinical application. Previous reports have shown the toxicology of GA. GA showed no serious CNS effects and caused no significant changes in spontaneous locomotor activity of mice (Zhao et al., 2010). However, GA has shown toxicity on pregnant rats and fetuses, and caused pectoral fin defect and lethal toxicity in zebrafish embryos in a dose-dependent manner (Zhao et al., 2010; Jiang et al., 2016). The LD_50_ of GA in mice was 45.993 mg/kg. In the beagle dog model, when GA (8 mg/kg) was injected intraperitoneally, the typical toxicological responses mainly included hydrostomia, astasia, and anepithymia (Guo et al., 2006). Most of all, long-term use of a high dose of GA led to damage to the kidney and liver in Sprague–Dawley rats (Qi et al., 2008). The symptoms of adverse reactions following GA injection administration include abdominal pain, phlebitis, and nausea in the phase IIa study (Yihebali et al., 2013). The phase II clinical trial of GA approved for the treatment of NSCLC has been terminated probably due to the high toxicity of GA, especially liver toxicity. Therefore, additional manners should be explored to improve reverse reaction while retaining the antitumor activity of GA, for example, precision drug delivery and chemical structure modification. GA was conjugated with unsaturated long-chain oleyl alcohol (OA) and self-assembled into NPs in water to prepare GA-OA@TPGS/NPs. The GA-OA@TPGS/NPs showed excellent stability, prolonged circulation, more precise targeting of tumor cells, and most importantly, lower toxicity (Wang et al., 2024). In the previous study, a new delivery nanoparticle containing both tumor-penetrating peptide (internalizing RGD peptide, iRGD) and EGFR single-domain antibody (sdAb) was constructed, the anti-EGFR-iRGD recombinant protein was modified on the surface of red blood cell membrane-coated nanoparticle (RBCm-NP), and GA was loaded. The new iE-RBCm-GA/PLGA NPs enhanced the diffusion ability of GA into cancer cells *in vitro*, increased stability and biocompatibility, and reduced side effects *in vivo* (Zhang et al., 2018). GA was loaded into a novel situ nanocomposite hydrogel vaccine system (Gel-NP@GA), along with a near-infrared (NIR) fluorescent dye, causing a sustained GA release to reduce toxicity reactions and enhance antitumor effects (Lei et al., 2025). The mesoporous polydopamine (MPDA) nanoparticles endowed with photothermal conversion capabilities could be used to deliver GA, and the GA-loaded GA@MPDA NPs significantly inhibited tumor growth and reduced the toxicity in vital organs (heart, liver, lung, spleen, and kidney) (Liu et al., 2024). Many studies have focused on the effect of chemical structure modification on the biological activity of GA, but little attention has been paid to the relationship between toxicity and chemical structure (Wang et al., 2009; Wang et al., 2011). We speculate that chemical modification may be a new avenue for the reduction in GA toxicity.

## 3 Gambogenic acid

**FIGURE 3:**
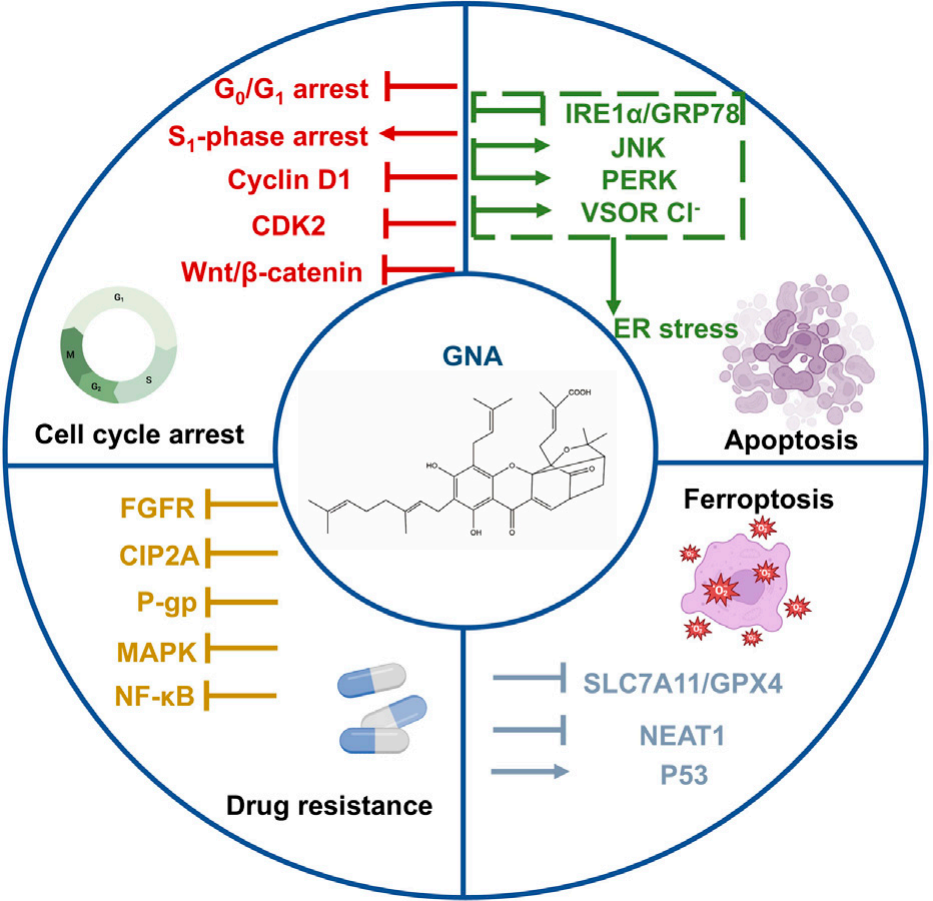
Antitumor mechanisms of gamboge active ingredient gambogenic acid.

GNA is another active component from gamboge with a structure similar to GA. GNA has more substantial antitumor effects and lower systemic toxicity than GA and exerts antitumor activity through several mechanisms, including the induction of apoptosis and ferroptosis, and cell cycle arrest (Sun et al., 2018). Here, we elaborate on these mechanisms in detail (Figure 3).

### 3.1 Antitumor mechanisms of GNA

#### 3.1.1 Apoptosis

GNA was found to increase the Bax/Bcl-2 ratio in a time-dependent manner and induce apoptosis through the mitochondrial pathway in human hepatoma HepG2 cells (Yan et al., 2012). GNA upregulated the expression of proapoptotic proteins and induced apoptosis in small-cell lung cancer (SCLC) cell lines (Huang et al., 2019). Endoplasmic reticulum (ER) stress exhibits a proapoptotic effect in tumor cells (Oakes, 2020; Albayrak et al., 2021). GNA induced ER stress by overproducing ROS, leading to the dissociation of inositol-requiring enzyme-1α (IRE1α) from glucose-regulated protein 78 (GRP78), which then activated JNK to trigger apoptosis in CRC cells (Zhao et al., 2020). GNA triggered ER stress through interaction with Aurora A in HCT116 cells, suppressing CRC. The phosphorylation of PERK and downstream PERK was observed in HCT116 cells. Therefore, the activation of ER stress may be mediated by promoting the PERK signaling pathway in addition to IRE1α (Wang et al., 2021). Moreover, GNA activated volume-sensitive chloride (VSOR Cl^−^) channels to trigger ER stress and eventually induced apoptosis in human NPC CNE-2Z cells (Su et al., 2019).

#### 3.1.2 Cell cycle arrest

In SCLC NCI-H446 cells, low doses of GNA blocked the cycle in the G_0_/G_1_ phase, whereas higher-dose concentrations induced S phase arrest (Huang et al., 2019). High concentrations of GNA significantly blocked the G_0_/G_1_ phase in CNE-1cells (Yan et al., 2011). GNA induced G_1_ phase arrest in lung cancer cells by promoting the degradation of GSK3β-dependent cyclin D1 and inhibiting CDK2 (Yu et al., 2012). Similarly, GNA reduced the level of cyclin D1 and blocked the G_0_/G_1_ cycle of CRC stem cells related to the Wnt/β-catenin signaling pathway (Li et al., 2022a).

#### 3.1.3 Ferroptosis

Ferroptosis is a novel cell-programmed death mediated by iron-dependent lipid peroxidation, characterized by the overload of iron, the accumulation of ROS, and lipid peroxidation (Wang M. et al., 2020; Wu et al., 2020). The inhibition of the cystine/glutamate antiporter (System xc^−^) is a disulfide-linked heterodimer composed of solute carrier family 7 member 11 (SLC7A11) and solute carrier family 3 member 2 (SLC3A2) (Dixon et al., 2012; Xu T. et al., 2019). GNA triggered ferroptosis in melanoma cells by decreasing lncRNA nuclear-enriched abundant transcript 1 (NEAT1) and downregulating levels of SLC7A11/glutathione peroxidase 4 (GPX4) (Wang M. et al., 2022). In addition, GNA activated the p53/SLC7A11/GPX4 signaling pathway, disrupted the oxidative stress balance, with increased ROS accumulation in TGF-β1-induced treated melanoma cells, and then triggered ferroptosis (Wang M. et al., 2020).

#### 3.1.4 Drug resistance

GNA potentiated the efficacy of erlotinib in inhibiting NSCLC cell proliferation by suppressing the fibroblast growth factor receptor (FGFR) signaling pathway. GNA and erlotinib synergistically inhibited HCC827 erlotinib-resistant (HCC827ER) xenograft growth *in vivo* (Xu et al., 2018). The overexpression of the cancerous inhibitor of protein phosphatase 2A (CIP2A) is related to resistance and tumor formation. GNA induced degradation of CIP2A and enhanced sensitivity to antitumor agents in hepatocellular carcinoma. However, the mechanisms of GNA to promote CIP2A degradation remain unclear and require further investigation (Yu et al., 2016). GNA potentiated the apoptotic effect of bortezomib in human myeloma MM.1S cells by regulating apoptosis-related proteins (Chen et al., 2017). In addition, GNA downregulated P-gp and P-gp-related proteins to reverse multidrug resistance in HepG2/ADR cells, probably by inhibiting the NF-κB and MAPK pathways (Xu Q. et al., 2019).

#### 3.1.5 Other mechanisms

GNA inhibited NF-κB signaling by downregulating p65 expression and suppressing the metastasis of bladder cancer cells (Zhou et al., 2020). Mei et al. (2014) demonstrated that GNA blocked the degradation of p62, leading to aberrant autophagic degradation that plays a pro-death role in GNA-mediated cell death.

### 3.2 Nanoscale drug delivery system

Efforts have been made to investigate novel GNA delivery systems to overcome the problems of GNA, including poor water solubility, high vascular irritation, and low bioavailability. Wang et al. (2021) prepared functional polydopamine nanoparticles to encapsulate and stabilize GNA, improving the bioavailability and tumor-targ e ting action of raw GNA. The curve ( ${AUC}_{0\to \infty }$ ) of the plasma drug concentration–time of polydopamine-coating GNA-loaded Zein nanoparticles was approximately 3.48-fold higher than that of GNA *in vivo* (Zha et al., 2020). PEGylated liposomes as delivery systems for GNA increased the local GNA concentration at the tumor site after tail vein injection and showed higher antitumor efficacy than GNA *in vitro*/*in vivo* (Tang et al., 2018). The relative bioavailability of GNA nanosuspensions prepared using the anti-solvent precipitation method was 263%, and it exhibited a longer t_1/2_ than the GNA solution (Yuan et al., 2016). Luo et al. (2015) prepared glyceryl monoolein-bearing cubosomes for GNA that showed higher AUC and C_max_ obtaining long-circulating colloidal delivery systems after intraperitoneal administration.

### 3.3 Toxicity and clinical application

GNA is a derivative formed by opening the pyran ring of GA and exerts less systemic toxicity than GA. GNA showed no effect on body weight in mice (Xu et al., 2018; Chen et al., 2020a). In the SCLC xenograft mice, no apoptotic cell death was observed in the lung, liver, kidney, spleen, or heart tissues after GNA treatment (Xu et al., 2018). Meanwhile, GNA possessed a liver-protective effect by attenuating the acetaminophen (APAP)-induced liver injury, inflammation, and apoptosis (Ding et al., 2021). GNA has not been used in the clinic alone despite its significant antitumor activity and hypotonicity. GNA has stronger antitumor effects and lower systemic toxicity than GA, but research and applications are limited. It is expected that GNA will receive more attention to exploration in future studies.

## 4 Other active ingredients

### 4.1 Isogambogenic acid

Iso-GNA is an isomer compound of GNA. Iso-GNA induced apoptosis-independent autophagic cell death by inhibiting the Akt-mTOR signaling pathway and overcame drug resistance caused by apoptosis deficiency in NSCLC (Yang et al., 2015). Iso-GNA promoted autophagy and apoptosis in glioma cells by activating the AMPK/mTOR pathway (Zhao et al., 2017). Furthermore, iso-GNA exhibited HUVEC migration *in vitro* and had antiangiogenic activities with less toxicity than GA *in vivo* (Yang et al., 2013).

### 4.2 Isomorellin and forbesione

Isomorellin and forbesione are caged polyprenylated xanthones isolated from gamboge. Isomorellin reduced cholangiocarcinoma (CCA) KKU-100 cell migration and invasion by downregulating FAK and inhibiting NF-κB signaling translocation (Hahnvajanawong et al., 2021). Moreover, isomorellin downregulated proteins that operate the G_0_/G_1_ phase, including cyclin D1, cyclin E, Cdk2, and Cdk4, and arrested the cell cycle in CCA cell lines (Hahnvajanawong et al., 2012). Forbesione inhibited the growth of CCA cell lines *in vitro*/*in vivo* by triggering S phase arrest and apoptosis through multiple pathways (Boueroy et al., 2016). Additionally, forbesione was found to synergistically exhibit antitumor effects with 5-FU in Ham-1 cells through apoptosis induction (Boueroy et al., 2017). Isomorellin and forbesione induced apoptosis by regulating the expression of apoptosis-related genes and proteins in CCA cell lines (Hahnvajanawong et al., 2010). In addition, isomorellin/DOX and forbesione/DOX combinations showed synergistic effects on CCA cells, but the same drug combinations did not show synergistic properties in human liver Chang cells. The result indicated that isomorellin and forbesione enhanced the antitumor effects of DOX and selectively inhibited the growth of CCA cell lines (Hahnvajanawong et al., 2014).

### 4.3 Extract of gamboge

The ethanolic extract of gamboge (EGG) upregulated E-cadherin expression to induce dose-dependent apoptosis in colon cancer cells. In addition, EGG reduced β-catenin to inhibit Wnt signaling, resulting in decreased cyclin D1 and matrix metalloproteinase (MMP)-7 (Wang W. et al., 2018).

## 5 Discussion

Natural products have evolved over a long period in nature, and TCMs have been used for thousands of years. Although there are many problems of active ingredients from TCMs, such as poor water solubility, poor stability, and low bioavailability, which limited the clinical application, preclinical research works showed that various active ingredients from TCMs have excellent antitumor abilities. Gamboge is a reddish yellow/orange-yellow colloidal resin secreted by *Garcinia hanburyi* Hook f., mainly from China, Cambodia, Thailand, Vietnam, India, and other tropical regions, and has been used to treat scrofula, carbuncle, and boils, which modern medicine considers to be inflammation or cancer. In this review, we summarized the anticancer effect and mechanism of the active ingredients from gamboge. GA prevents the development of tumors by inducing apoptosis, regulating cell autophagy, blocking the cell cycle, restricting cell metastasis, impeding angiogenesis, and reversing drug resistance. GNA has more substantial antitumor effects and less systemic toxicity than GA and exerts antitumor activity through several mechanisms, including the induction of apoptosis and ferroptosis, cell cycle arrest, and drug resistance reversion. Iso-GNA primarily induces autophagy and inhibits angiogenesis. Isomorellin can induce cell cycle arrest and suppress tumor cell migration. Forbesione promotes cancer cell apoptosis to inhibit tumor growth. Meanwhile, the side effect of gamboge limits the clinical application, especially GA. Previous studies have mentioned that a high dose of GA can impair kidney and liver function. GA also affected pregnant rats and fetuses in a dose-dependent manner. GNA/iso-GNA exerted lower toxicity *in vitro*/*in vivo* than GA, but the specific mechanisms remain unknown because of the lack of specialized toxicity tests of GNA/iso-GNA. GNA/iso-GNA differs from GA in terms of the structure on the open ring of its pyran. We presume that the poisonousness of GA is possibly due to the presence of its pyran ring. Therefore, the reverse effects might be improved by changing the chemical structure of GA. Moreover, almost no studies have elaborated on the toxicity of other components; the side effects of these compounds with excellent antitumor activity should be explored in the future.

The reverse effects of GA might have led to the failure of its clinical trials, and the clinical superiority assessment of GA was disrupted due to the rapid metabolism and short half-life (Wang et al., 2024). The clinical translation of GA is limited by these challenges. As a result, we suppose that more delivery systems or chemical modifications probably promote the development. Reducing the dose of administration, increasing dosing intervals, or combining with other chemotherapy drugs may decrease the toxicity of GA. Compared to GA, GNA showed less toxicity and even protective effects on the liver. GNA has a higher potential for clinical application.

When compared to other anticancer agents, the active ingredients of gamboge have a number of advantages over them. For example, the current approaches for treating GBM are surgery, radiotherapy, and chemotherapy using TMZ. However, the primary obstacle remained the emergence of TMZ resistance. In a previous study, GA caused stronger apoptosis in TMZ- and IR-resistant cells, and the combination of GA and TMZ or IR enhanced therapeutic efficacy (Sun et al., 2024). GA has been shown to be more cytotoxic than CDDP in NSCLC cell lines *in vitro*; GA also sensitized NSCLC cells to CDDP to inhibit tumor growth *in vivo* (Wang L. H. et al., 2014). Moreover, GNA exhibited potent inhibitory activities in CDDP-resistant NSCLC cells (Shen et al., 2020), and GNA potentiated the therapeutic efficacy of erlotinib on NSCLC (Xu et al., 2018). The application of gamboge may solve the challenges of drug resistance in the clinic and provide more therapeutic approaches for patients suffering from refractor and recurrent cancers.

## 6 Conclusion

In this review, we summarized the research on antitumor properties and mechanisms of gamboge’s active xanthone ingredients. Evidence suggests that the active components of gamboge have various antitumor activities *in vitro* and *in vivo*, including triggering cell apoptosis, inducing cell cycle arrest, and inhibiting cell invasion and migration. Several novel drug delivery systems are listed simultaneously, such as GA-loaded micelles, nanoparticles, and GNA-encapsulated nanoparticles and liposomes. Moreover, the antitumor mechanisms of these compounds still need to be further explored to develop more antitumor agents. There was little development in exploring the structure of the xanthone moiety of gamboge ingredients. Therefore, the commonalities of xanthone moieties and the role of the structure in treating cancers need to be explored in the future. In conclusion, the development of active ingredients from gamboge will attract more and more attention, especially in clinical applications.

## Glossary

5-FU: 5-fluorouracil
APAP: attenuating the acetaminophen
BMI1: B-cell-specific Moloney leukemia virus insert site 1
CCA: cholangiocarcinoma
CDDP: cisplatin
CFDA: China Food and Drug Administration
cFLIP: cellular FADD-like apoptosis regulator
CIP2A: cancerous inhibitor of protein phosphatase 2A
CML: chronic myeloid leukemia
CQ: chloroquine
CRC: colorectal cancer
DIO-1: death-inducer obliterator
DOX: doxorubicin
EGG: ethanolic extract of gamboge
ER: endoplasmic reticulum
ERα: estrogen receptor alpha
FGFR: fibroblast growth factor receptor
FUS: focused ultrasound
GA/PLGA-CMB: GA-loaded nanobubble–microbubble complexes
GA: gambogic acid
GAL: liposomal formulation of GA
GBM: glioblastoma
Gem: gemcitabine
GNA: gambogenic acid
GPR108: G protein-coupled receptor 108
GPX4: glutathione peroxidase 4
GRP78: glucose-regulated protein 78
GSCs: glioma stem cells
HCC827ER: HCC827 erlotinib-resistant
HUVECs: human umbilical vein endothelial cells
IRE1α: inositol-requiring enzyme-1α
iRGD: internalizing RGD peptide
iso-GNA: isogambogenic acid
JNK: C-Jun N-terminal kinase
LKB1: liver kinase B1
MDM2: murine double minute 2
MM: multiple myeloma
MMP: matrix metalloproteinase
mPEG: monomethoxy-poly(ethylene glycol)
NEAT1: nuclear-enriched abundant transcript 1
NPC: nasopharyngeal carcinoma
NSCLC: non-small-cell lung cancer
OA: oleyl alcohol
PCNA: proliferating cell nuclear antigen
PEI: polyethylenimine
RBCm-GA/PLGA NPs: GA-loaded biomimetic nanoparticles
RBCm-NP: red blood cell membrane-coated nanoparticle
ROS: reactive oxygen species
RRM2: ribonucleotide reductase subunit-M2
SCLC: small-cell lung cancer
sdAb: single-domain antibody
SLC3A2: solute carrier family 3 member 2
SLC7A11: solute carrier family 7 member 11
SPEG/HA/CSO-SS-HEX/Fe3O4/GA: redox/pH dual-responsive and magnetic targeted hybrid multifunctional complex micelles
**System xc** ^**−**^: the cystine/glutamate antiporter
TCM: traditional Chinese medicine
TfR: transferrin receptor
TNBC: triple-negative breast cancer
TNF: tumor necrosis factor
TRAIL: TNF-related apoptosis-inducing ligand
TrxR: thioredoxin reductase
VEGF: vascular endothelial growth factor-A
VEGFR2: vascular endothelial growth factor receptor 2
VSOR Cl−: volume-sensitive chloride
WSC: water-soluble chitosan
